# Photothrombosis-Induced Infarction of the Mouse Cerebral Cortex Is Not Affected by the Nrf2-Activator Sulforaphane

**DOI:** 10.1371/journal.pone.0041090

**Published:** 2012-07-23

**Authors:** Michelle J. Porritt, Helene C. Andersson, Linda Hou, Åsa Nilsson, Marcela Pekna, Milos Pekny, Michael Nilsson

**Affiliations:** 1 Center for Brain Repair and Rehabilitation (CBR), Institute of Neuroscience and Physiology, the Sahlgrenska Academy, University of Gothenburg, Gothenburg, Sweden; 2 Sahlgrenska University Hospital, Gothenburg, Sweden; Albany Medical College, United States of America

## Abstract

Sulforaphane-induced activation of the transcription factor NF-E2 related factor 2 (Nrf2 or the gene Nfe2l2) and subsequent induction of the phase II antioxidant system has previously been shown to exert neuroprotective action in a transient model of focal cerebral ischemia. However, its ability to attenuate functional and cellular deficits after permanent focal cerebral ischemia is not clear. We assessed the neuroprotective effects of sulforaphane in the photothrombotic model of permanent focal cerebral ischemia. Sulforaphane was administered (5 or 50 mg/kg, i.p.) after ischemic onset either as a single dose or as daily doses for 3 days. Sulforaphane increased transcription of Nrf2, Hmox1, GCLC and GSTA4 mRNA in the brain confirming activation of the Nrf2 system. Single or repeated administration of sulforaphane had no effect on the infarct volume, nor did it reduce the number of activated glial cells or proliferating cells when analyzed 24 and 72 h after stroke. Motor-function as assessed by beam-walking, cylinder-test, and adhesive test, did not improve after sulforaphane treatment. The results show that sulforaphane treatment initiated after photothrombosis-induced permanent cerebral ischemia does not interfere with key cellular mechanisms underlying tissue damage.

## Introduction

Stroke represents one of the most costly and long-term disabling conditions in adulthood worldwide. The majority of stroke patients suffer from a permanent cerebral ischemia, as less than 2% of patients arrive at hospital within the therapeutic thrombolysis time window of 4.5 hours [1], [2]. Despite this, most experimental stroke neuroprotection studies are performed in transient stroke models where reperfusion occurs after a short period of occlusion [3], [4].

A key pathological feature of ischemic stroke and many other neurological diseases is oxidative stress. This is due to an excessive production of reactive oxygen species (ROS), a decreased cellular defense capability or both. The transcription factor NF-E2 related factor 2 (Nrf2) is a key regulator of the cellular antioxidant defence. Cellular stress results in translocation of Nrf2 to the nucleus and triggers the transcription of ARE-mediated gene products, such as heme oxygenase (Hmox1), NAD(P)H quinone oxidoreductase (Nqo1), glutamate–cysteine ligase catalytic subunit (GCLC), glutathione peroxidase (GPx), glutathione S-transferase A4 (GSTA4), and glutathione s-transferase (GST) [5]–[7]. These maintain redox homeostasis via antioxidant, detoxification and anti-inflammatory properties. Neuroprotective effects following cerebral ischemia has been demonstrated previously to be associated with altered expression of these genes [8]–[11].

The Nrf2 pathway can be activated by different phytochemicals such as sulforaphane. Sulforaphane is an isothiocyanate obtained from cruciferous vegetables. It is a potent inducer of cytoprotective proteins that acts as an indirect antioxidant by inducing Nrf2-dependent gene expression [12]. In models of transient cerebral ischemia and traumatic brain injury, activation of the Nrf2 system by a single dose of sulforaphane leads to neuroprotection [13], [14]. Repeated administration of sulforaphane ameliorates motor deficits and cortical cell death in a model of subarachnoid haemorrhage [15] and repeated exposure of cultured astrocytes to sulforaphane has an additive effect on the Nrf2 mediated gene and protein expression and protection against superoxide-induced damage [16].

The majority of *in vivo* studies report neuroprotection when the Nrf2-system is activated prior to or very shortly after injury [14], [17]–[19]. Therefore, the goal of this study was to investigate the neuroprotective effect of the Nrf2 system activation by a single or repeated dosage of sulforaphane following a permanent ischemic stroke in adult mice. Permanent focal ischemia was induced by photothrombosis, a model that has the advantage of being highly reproducible with respect to the infarct location and size. The photothrombotic stroke generates an irreversibly damaged ischemic core within the cortex surrounded by a small penumbral region [20], [21]. Sulforaphane or vehicle was injected as a single intraperitoneal injection 15 min after ischemic onset or once daily for three days. The effect of sulforaphane on the outcome was evaluated by measuring infarct volume and motor and sensory function, as assessed by beam-walking, cylinder-test, and adhesive test. In addition, the effect on reactive gliosis was determined by quantifying activated microglia, astrocytes and proliferating cells.

## Materials and Methods

### Animal Preparation

Adult male C57BL/6 mice weighing approximately 25 g (Charles River, Germany) were used. A total of 62 mice were randomly allocated to drug treatment or vehicle and underwent stroke induction with 61 animals included in the final analysis of infarct volume and cell distribution. Pilot experiments indicated that the variability in infarct size was 18% of the mean infarct volume. Thus, a power calculation showed that a minimum of eight animals were required per cohort to detect the same 30% reduction of infarct volume observed by Zhao and colleagues, with 80% power and an α of 0.05 [14]. One mouse (50 mg/kg sulforaphane group) died overnight; a post mortem revealed no obvious cause of death such as haemorrhage, but the death is conceivably to have resulted from the stroke (total experiment mortality rate of 1.6%). The animals were housed under standard conditions on a 12 h light/12 h dark cycle with food and water *ad libitum*. All experimental protocols were approved by the Animal Ethics Committee of the University of Gothenburg (146–2008) and performed according to approved NIH animal care guidelines.

### Rose Bengal Photothrombotic Stroke Induction

Cortical photothrombosis was induced by the Rose Bengal technique [22], [23]. The surgeon was blinded to the assigned randomly allocated drug treatment. Anaesthesia was induced with Isoflurane in air and oxygen (1∶1) initially at 2.5% then reduced to 1% during the surgical procedure. Body temperature was monitored by a rectal probe and maintained at 37°C using a homeothermic control unit (Harvard Apparatus, USA). Anaesthetized mice were placed in a stereotaxic frame, the scalp incised and the laser positioned A0.5 mm and L2.7 mm relative to Bregma [24]. Rose Bengal (100 µl, 10 mg/ml solution in sterile saline) was injected intraperitoneally. After 5 min, the laser (Cobolt, 50 mW, 561 nm) was illuminated for 10 min. The scalp was sutured and the mice left to recover in a 22°C chamber for 1 h prior to returning to their home cage. Mice were provided a heat mat and a dish of moist food in their home cage for the duration of their post-stroke survival. The animals temperature was pegged at 37^o^C and all surgical times were recorded. There was no difference in animal weight, temperature or duration of surgery between the different cohorts.

### Sulforaphane Administration

Sulforaphane (Alexis Biochemicals Enzo Life Science, Switzerland) was dissolved in DMSO and then diluted to 0.5 mg/ml with either corn oil or sterile saline (final DMSO concentration 1%). For behavioural and histological analyses, animals were injected intra-peritoneally with 5 mg/kg sulforaphane diluted in corn oil or 50 mg/kg diluted in sterile saline 15 min after the ischemic injury, and sacrificed 24 h later. For further histological and behavioural analyses, an additional set of animals were injected with 5 or 50 mg/kg sulforaphane (in sterile saline) 15 min, 24 h and 48 h after the ischemic injury and sacrificed 72 h later. A cohort of naïve mice was injected with a single dose of sulforaphane (5 or 50 mg/kg) or vehicle and sacrificed 24 h later for RNA isolation.

### BrdU Administration

To detect proliferating cells, the mice were injected with bromodeoxyuridine (BrdU). BrdU (Roche Diagnostics, Mannheim, Germany) was dissolved in 0.9% NaCl and filtered with a 22 µm syringe filter. Intraperitoneal injections of BrdU (50 mg/kg) were administered 15 min, 24 h and 48 h after the ischemic injury.

### Evaluation of Neurological Deficits - Behavioural Testing

All sensorimotor tests were performed at the end of the dark cycle of the animal housing, with conditions consistently maintained across examinations. All behavioural tests had objective outcome measures and the assessors were blind to the treatment group of each animal. Mice were acclimatised to the behavioural tests prior to the commencement of stroke induction and drug treatment, and the measurements taken 24 h prior to stroke were used as their respective baseline. The animals were then tested 24 and 72 h after stroke onset.

#### Beam walking

The distance and time taken to walk across a 60 cm long beam of 1.2 cm square diameter and a round 1cm diameter, suspended 60 cm over the bench was recorded for each animal.

#### Cylinder test

The method of Schallert and colleagues [25], [26] was used with minor modifications. A glass cylinder, 12 cm in diameter, was used as it allowed mice to stand comfortably on the base with only 1–2 cm in front and behind thus encouraging rearing. The number of times the mice reared, and the forepaw used to make first contact in 2 min was recorded.

#### Adhesive test

The test was performed in the home cage of the mice, with the bedding removed. Small adhesive stickers were placed onto the front paws of the mice and the length of time taken to remove it was recorded. This test was repeated twice on each occasion and the mean of both scores was used in the analysis. The mice were given a maximum of 3 min to perform this task.

### Animal Perfusion and Tissue Dissection

Twenty four hours and 72 h after stroke induction the animals were sacrificed by an overdose of pentobarbital and transcardially perfused with saline, then Histofix (Histolab Products AB, Gothenburg, Sweden). The brains were extracted and post-fixed overnight in the same fixative prior to cryoprotection in 30% sucrose. The brains were frozen in isopentane on dry ice and stored at −80^o^C.

### Histology

Frozen sections (25 µm) were cut in the coronal plane and thaw mounted onto Superfrost plus slides. Sections were stored at −20°C. Haematoxylin and eosin (H+E) staining was performed to delineate the ischemic infarct.

### Infarct Volume Quantification

Digital images containing of the H+E sections were produced. The ischemic lesion was clearly delineated as a region of pallor within the somatosensory cortex. A calibration standard was included in each image. The assessor, blind to drug treatment allocation and survival interval, outlined the infarct with Image J (NIH, version 1.41o) on nine standard coronal planes from each brain (from Bregma in mm, 1.98, 1.54, 0.98, 0.5, 0.02, −0.58, −1.06, −2.06, −2.54). Infarct volumes were derived by calculating the average infarct area between slices and multiplying by the distance between slices. Edema corrections were calculated by dividing the ipsilateral by the contralateral hemisphere volume.

### Detection of Reactive Glia and Dividing Cells

For immunohistochemical detection of glial and proliferating cells, sections were rinsed 3 times in TBS prior to blocking endogenous peroxidase activity with 0.6% H_2_O_2_. Sections were rinsed again in TBS for 2×10 min and, for detection of BrdU the DNA was denatured by incubating the sections in 2M HCl for 30 min in 37°C, and thereafter 10 min in 0.1 M borate buffer, pH 8.5. All sections were then incubated with blocking solution for 30 min (TBS with 0.1% Triton X-100 and 3% normal donkey serum) before incubating with primary antibody in blocking solution over night at 4°C. Dilutions of primary antibodies used were as follows: mouse anti-BrdU (1∶200, Roche), polyclonal rabbit anti-GFAP (1∶500, Dako, A/S, Denmark), and rabbit anti-Iba1 (1∶500, Wako Pure Chemical Indust. VA, USA). After rinsing with TBS, sections were incubated for 2 h in biotinylated donkey anti-mouse IgG secondary antibody or biotinylated donkey anti-rabbit IgG secondary antibody (both 1∶1000, Jackson ImmunoResearch, West Grove, PA) followed by avidin-biotin-peroxidase complex for 1 h (Vector Laboratories, Burlingame, CA). The sections were rinsed in TBS and the peroxidase detected by incubation in 3, 3′-diamino-benzidine solution for 5 min (0.25 mg/mL DAB; Saveen Werner AB, Malmö, Sweden) in the presence of 0.01% H_2_O_2_ and 0.04% nickel ammonium sulphate. Sections were rinsed in water then dehydrated. Specificity of staining was determined by omission of the primary antibodies.

### Quantitation of Reactive Glia and Dividing Cells

Quantification was performed by an observer blinded to treatment group. Three sections (25 µm, 200 µm apart) from 8–10 animals per group were viewed by bright field microscopy and images were captured with a Nikon Optishot 2 and a microscope equipped with a Hamamatsu C5810 colour chilled 3CCD camera. The sections, from the middle of the infarct core, represented 0.9 to 0.5 mm from Bregma. The number of positive cells was determined in a 300 µm wide area of cortex from each of the following 3 regions: peri-infarct, transition, and infarct core. The transition zone was classified as 150 µm either side of the infarct core boundary (StereoInvestigator, MicroBrightField Inc., Colchester, VT, USA). The number of cells observed was divided by the area sampled and is presented as a density.

### Quantitative PCR Analysis of Nqo1 Gene Expression

Tissue samples (2 mm by 2 mm blocks) of parietal somatosensory cortex (n = 3) from vehicle and sulforaphane (5 or 50 mg/kg) treated mice, were snap-frozen in liquid nitrogen 24 h after treatment. The tissue blocks were thawed by the addition of Buffer MRL (Qiagen) and total RNA extracted. RNA concentration was measured with a Nanodrop. cDNA was synthesized from 10 µl of mRNA from the total RNA extraction. The reaction buffer consisted of First Strand Buffer x1, 10 mM dithiothretiol (DTT) and 5U/µl SuperScript^TM^ II Reverse Transcriptase (Invitrogen) and 1U/µl Protector RNase Inhibitor, 20 pmole/µl Hexanucleotide Mix and 0.25 mM of each dNTP and Li-Salt (Roche Diagnostics). The reverse transcriptase reaction was performed on a PTC-200 Peltier Thermal Cycler (MJ Research) at 22°C for 10 min, 42°C for 45 min and 99°C for 3 min.

Inventoried TaqMan® Gene Expression Assays were purchased from Applied Biosystems. The exact primer sequences are unknown; according to Applied Biosystems’ policy. Primers used were; NAD(P)H-dehydrogenase quinone 1/Nqo1, Assay ID Mm00500821_m1, heme oxygenase/Hmox1, Assay ID Mm00516005_m1, glutamate–cysteine ligase catalytic subunit/Gclc, Assay ID Mm00802655_m1, glutathione S-transferase A4/GSTA4, Assay ID Mm00494803_m1, nuclear factor (erythroid-derived 2)-like 2/Nfe2l2, Assay ID Mm00477784_m1 and Polymerase (RNA) II (DNA directed) polypeptide A (mapped)/Polr2a, Assay ID Mm00839493_m1. To avoid DNA amplification, the primer products span exon-exon junctions. qPCR reactions were performed according to manufacturer’s instructions. The results were analyzed with SDS 2.3 software (Applied Biosystems) and the relative quantity was determined using the ΔΔC_T_ Method [27] with untreated brain as calibrator and Polr2a as the endogenous control.

### Statistical Analysis

Data is presented as the mean ± SD. Differences between means were assessed by ANOVA one or two tailed with *p*-values ≤ 0.05 considered significant.

## Results

### Infarct Volume is Unaltered by Sulforaphane Treatment

**Figure 1:**
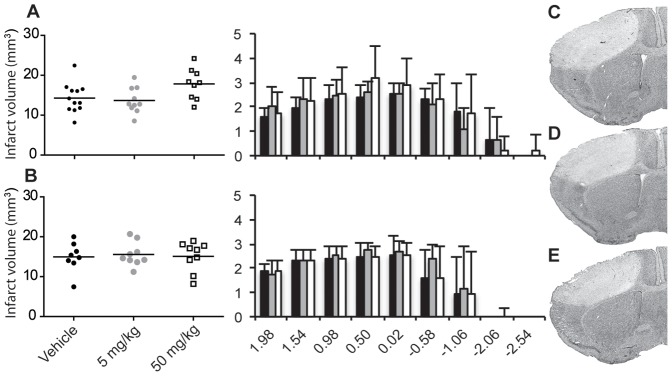
Infarct volume presented as scattergrams and as their distribution across in anterior and posterior plane at A) 24 h and B) 72 h after ischemia. Infarct volume and distribution did not change with time. Single or repeated doses of sulforaphane did not protect as infarct volume did not differ in comparison to vehicle treated animals (mean±SD; *n* = 8–13, one way ANOVA 24 h p = 0.38, 72 h p = 0.93; solid black vehicle, solid gre 5 mg/kg sulforaphane, open box 50 mg/kg sulforaphane). Coronal images of representative cortical infarcts at 0.5 mm from Bregma 72 h after onset in C) vehicle, D) 5 mg/kg sulforaphane and E) 50 mg/kg sulforaphane treated animals.

The infarct volume at 24 and 72 h after stroke was determined on 25 µm haematoxylin and eosin stained coronal sections (Fig. 1 C-E). The two groups of vehicle-treated animals (saline or corn oil) were similar, so their results were pooled and are presented as a single control group. A one-way ANOVA (with Tukey post-hoc comparison) was used to test for the neuroprotective effect of sulforaphane on infarct volume. The infarct volume at 24 hours post ischemic onset did not differ across the three groups (*F* (2, 28)  =  3.673, *p* = 0.862). The infarct volume and the distribution over the rostral caudal plane did not differ between vehicle and sulforaphane treated groups (Fig. 1A). In mice that received daily sulforaphane injections (5 or 50 mg/kg) and were sacrificed 72 h after ischemia, a one way ANOVA (post-hoc Tukey) determined that infarct volumes did not differ from the control (vehicle-treated) mice, between the sulforaphane-treated groups (*F* (2, 23)  =  0.0723, *p* = 0.798) (Fig. 1B). The 24 h and 72 h time point infarct volumes did not differ.

### Sulforaphane Induces Up Regulation of Nqo1 Expression

**Figure 2:**
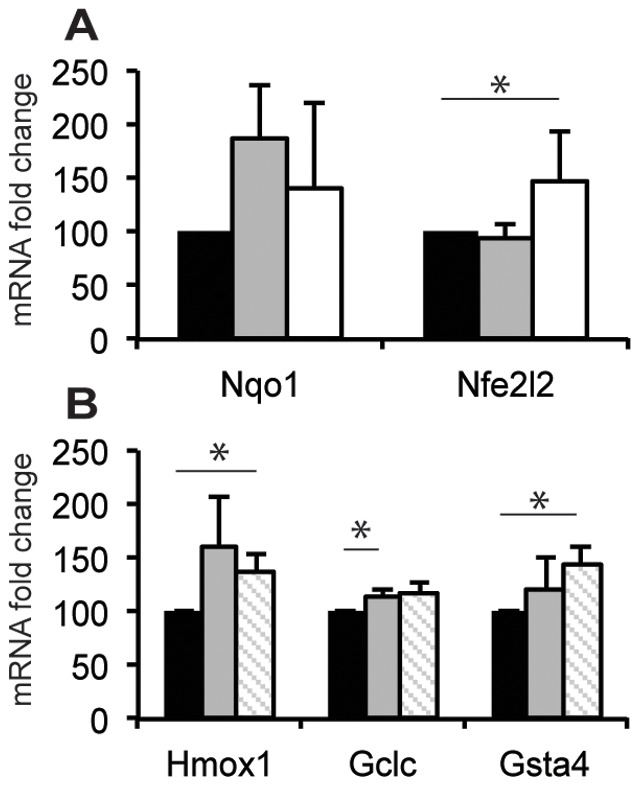
Messenger RNA levels of Nrf2 related gene products. **A**) Nqo1 and Nrf2 (Nfe2l2) expression in brain of naïve mice 24 h after treatment with sulforaphane or vehicle. Nrf2 is significantly increased by 5 mg/kg sulforaphane (mean±SD; *n* = 3, *p≤0.05; solid black box vehicle, solid grey box 5 mg/kg sulforaphane, open box 50 mg/kg sulforaphane). **B**) There was no additive effect of sulforaphane on stimulation of the Nrf2 system. Hmox1 and Gsta4 expression was significantly greater in animals 12 h after ischemia and 5 mg/kg sulforaphane treatment. GCLC treatment was increased by ischemia alone. (mean±SD; *n* = 3, ***p≤0.0001; solid black box sham and vehicle, solid grey box ischemia alone, open box grey diagonal stripes ischemia and 5 mg/kg sulforaphane).

To confirm that sulforaphane treatment activated the Nrf2 pathway of gene expression, the transcript of the Nrf2-mediated gene Nqo1 and Nfe2l2 was analysed 24 h after treatment of naïve mice. Single sulforaphane administration increased the expression of Nqo1 mRNA and significantly (*p*≤0.05) increased Nfe2l2 in the brain compared to vehicle treated animals (Fig. 2A).

The expression of Hmox1, Gclc and Gsta4, all phase II enzymes with Nrf2 related expression, was determined 12 hours after treatment with vehicle, photothrombotic ischemia alone or photothrombotic ischemia and low dose (5 mg/kg) sulforaphane. Hmox1 expression was significantly increased in animals with ischemia and sulforaphane (*p*≤0.05) compared to vehicle treated mice. Sulforaphane treatment did not result in a greater Hmox1 response than stroke alone. A one-way ANOVA determined Gsta4 mRNA expression was similar to Hmox1. Gsta4 only significantly increased in stroke combined with sulforaphane treatment compared to vehicle (*p*≤0.05). The other Nrf2 related gene, Gclc, was significantly increased after ischemia compared to vehicle (*p*≤0.05). There was no further increase in expression compared to ischemia alone when sulforaphane was administered.

### The Effect of Sulforaphane Treatment on Motor Function

**Figure 3:**
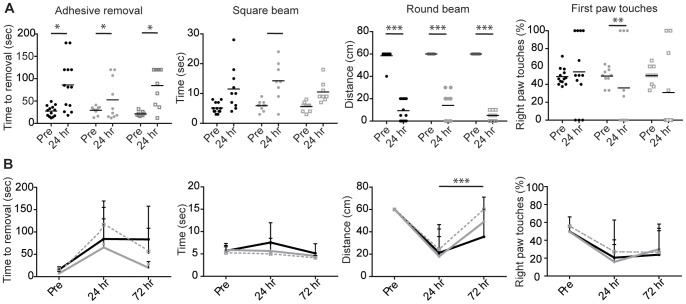
The motor function of the mice was examined prior to, 24 and 72 h after ischemic onset. **A**) 24 h after ischemia, all mice demonstrated significant functional deficits that were not affected by sulforaphane treatment. (mean±SD, *n* = 8–12, t-test or one way ANOVA *p≤0.05, **p≤0.001, ***p≤0.0001; solid black circle vehicle, solid grey circle 5 mg/kg sulforaphane, open box 50 mg/kg sulforaphane). **B**) 72 h after ischemia, mice treated with sulforaphane were able to complete the round beam task compared to vehicle. All other behavioural outcome measurements had returned to pre stroke baseline levels. (mean±SD, *n* = 8–12, solid black line vehicle, solid grey line 5 mg/kg sulforaphane, dashed grey line 50 mg/kg sulforaphane, *** F(2,48) = 53.21, *p*≤0.0001).

To examine whether sulforaphane influenced the functional deficit after ischemia, sensorimotor function was assessed. The two groups of vehicle-treated animals (saline or corn oil) were identical, so their results were pooled and are presented as a single control group. Paired t-tests confirmed that at twenty four hours after ischemia all animals spent a longer time crossing the square beam (*p*≤0.05), completed less distance on the round beam (*p*≤0.0005), reared less in the cylinder (*p*≤0.01) and took longer to identify and remove the adhesive sticker from their paws (*p*≤0.05), than they did to their respective baseline values (Fig. 3A). Sulforaphane treatment did not diminish the functional deficit at 24 h (Fig. 3A). In mice followed for 72 h, the initial deficit at 24 h post-ischemia was observed however all animals had regained functionality of their right forepaw and all the values returned to pre-stroke baseline levels at 72 h (Fig. 3B). A two way ANOVA (with Bonferroni multiple comparisons) revealed that there is a significant difference between vehicle and 5 mg/kg sulforaphane treatment at 72 h (*F* (2, 48)  =  19.55, *p*≤0.0001).

### The Effect of Sulforaphane on Reactive Gliosis

**Figure 4:**
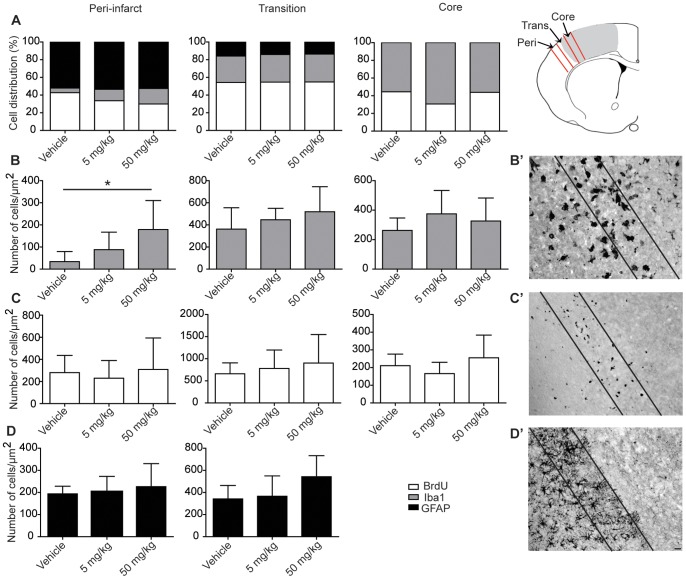
Cell proliferation and glial cell distribution determined by immunostaining 72 h after infarction. The bar graphs (mean±SD) represent the cell distribution in the peri-infarct, transition and core regions **A**) the percentage of total cell number per region and **B-D**) the number of cells/µm^2^, **B’**) activated microglia (Iba1), **C’**) proliferating cells (BrdU) and **D’**) reactive astrocytes (GFAP). No GFAP positive cells were observed in the core region. Sulforaphane treatment only altered the microglial cell number in the peri-infarct region compared to vehicle treatment (F(2,15) = 4.76, *p*≤0.025) (*n* = 5–9 in each group, solid black box GFAP astrocytes, solid grey box Iba1 microglia, open box BrdU proliferating cells).

In response to ischemic injury, glial cells become activated and proliferate [28]. After photothrombotic infarction, increased GFAP expression was observed in hypertrophied reactive astrocytes. Activated astrocytes were observed throughout the entire ipsilateral cortex (Fig. 4D’) at 72 h with the greatest density within the peri-infarct region, whilst no GFAP expressing astrocytes were found within the ischemic core (Fig. 4A, D). The GFAP expression was comparable in the sulforaphane-treated groups with no significant difference to the vehicle-treated group in the peri-infarct (*p* = 0.082) and transition region (*p* = 0.73).

In contrast to reactive astrocytes, activated microglia/macrophages and proliferating cells were confined to the transition and core regions of the ischemic lesion. Ischemia induced a robust activation of microglia/macrophages and proliferating cells 72 h after injury, as determined by antibodies against Iba1 (Fig. 4B’) and BrdU (Fig. 4C’). Seventy-two hours following injury, the density of Iba1 expressing cells was greatest in the transition and core regions compared to the peri-infarct (Fig. 4A, B). Within the peri-infarct there were significantly more Iba1 microglia cells in the 50 mg/kg mice than in vehicle treated mice (F (2,15) = 4.76, *p*≤0.025). The distribution and expression of Iba1 was comparable in the sulforaphane-treated groups and was not significantly different to vehicle-treated group in either the transition or ischemic core (one way ANOVA; transition *p* = 0.42 and ischemic core *p* = 0.582).

Proliferating cells were detected as round nuclei with BrdU immunostaining (Fig. 4C’). The greatest density of BrdU stained nuclei was in the transition area. The peri-infarct area and the infarct core had similar densities of BrdU positive cells (Fig. 3A, C). Sulforaphane treatment had no effect on the number of BrdU positive cells (one way ANOVA; peri-infarct p = 0.68, transition *p* = 0.61 and ischemic core *p* = 0.08).

## Discussion

Results from the present study show that sulforaphane treatment initiated after photothrombosis-induced permanent cerebral ischemia did not interfere with key cellular mechanisms underlying tissue damage. There was no reduction in the infarct volume and no difference in the functional deficit, or the extent of reactive gliosis and cell proliferation neither after administration of a single dosage of sulforaphane 15 minutes after the onset of ischemia nor after its repeated daily administration. Sulforaphane has been shown to be neuroprotective in different CNS injury models, including cerebral ischemia in rats, through activation of the redox-sensitive Nrf2 pathway [13]–[15]. Nqo1 is a multi-protective phase II enzyme under direct transcriptional control by Nrf2. [5], [29], [30]. Our results confirmed that single dose sulforaphane treatment was sufficient to activate the Nrf2 system in the CNS as demonstrated by increased Nqo1 expression in the parietal cortex 24 h after treatment in naïve mice.

After photothrombosis, the infarct volume was maximal at 24 h post stroke with no expansion observed at 72 h. In contrast to Zhao and colleagues, who reported that 5 mg/kg sulforaphane reduced infarct size by 30% when given 15 min after a transient middle cerebral artery occlusion in rats [14], we did not observe any difference in infarct volume with 5 or 50 mg/kg sulforaphane using a single dose in mice. Similarly, whereas repeated treatment with sulforaphane shortly after subarachnoid haemorrhage was neuroprotective in rats [15], repeated administration in the present study did not confer neuroprotection from photothrombotic ischemia. Focal ischemia in rats selectively up-regulates ARE-mediated gene expression. ARE mediated gene expression itself is mediated by Nrf2. This in conjunction with other ischemic cascade processes such as mitochondrial inhibition, oxidative stress and glucose deprivation all promote the activity of Nrf2 [31]–[33]. We hypothesize that the sulforaphane treatment starting 15 min after ischemia induction did not provide any further stimulation of the Nrf2 system in addition to the activation triggered by the ischemia alone.

Functional disabilities following ischemic stroke are often profound and therefore it is of fundamental importance to study changes in animal behaviour after ischemia. Specific tests on forepaw motor function demonstrated that photothrombotic infarction results in animals losing fine motor control of their paw. The mice spontaneously recovered use of their forepaw quickly (within 72 h) after infarction. In our study, single and repeated administration of sulforaphane did not affect the functional deficit at 24 h or the speed of functional recovery. However, as only two time points for the evaluation were used in our study and all the mice fully recovered by 72 h, we cannot exclude that the sulforaphane treatment affected the functional deficit between these time points.

The photothrombotic infarct is associated with extensive glial cell activation and recruitment of different neural cells in the areas surrounding the ischemic region [34]. Reactive gliosis constitutes a complex cellular response to different types of injuries and stress to the CNS and the evolving cellular reactions can be followed through the up-regulation of GFAP in astrocytes and Iba1 in microglia [35]. Interestingly, involvement of the Nrf2 system in the underlying mechanisms of glial cell activation has been proposed since Nrf2-deficient mice demonstrate widespread astrogliosis throughout the CNS [36]. However, in the present study, activation of the Nrf2 system by repeated sulforaphane treatment did not alter the number of BrdU, GFAP and Iba1 positive cells compared to vehicle injection, suggesting that at the time points and concentrations used, sulforaphane did not interfere with the mechanisms involved in the activation of glial cells or cell proliferation.

The photothrombotic model used in the present study induces a much smaller penumbra compared to the transient MCAO model where the collateral blood flow is more prominent [37]. This, together with the small volume of infarcted cortical tissue resulting from the photothrombosis injury, might result in the generation of relatively smaller amounts of ROS compared to the MCAO and may explain why we did not observe any neuroprotection after sulforaphane administration. The photothrombotic stroke model was chosen for the current study for the reason that it is highly reproducible regarding location and size, generating small infarcts limited to the cortex with a similar cellular response as the MCAO model [38], [39]. The model generates an irreversibly damaged ischemic core surrounded by a small penumbral region [20], [21]. Importantly, neuroprotection has previously been achieved in the photothrombotic model by i.e. the free radical scavenger 21-aminosteroid, NMDA and GABA_A_ receptor antagonists, the allosteric modulators lubeluzole and chlormethiazole and the calcium channel blocker flunarizine [40]–[42]. In contrast, MK801 and nimodipine were shown to be neuroprotective in other models of cerebral ischemia but failed to be neuroprotective in photothrombotic ischemia [40]–[43]. Based on our findings, sulforaphane is another compound the neuroprotective effects of which are model dependent.

In summary, the results from the present study demonstrate that sulforaphane treatment initiated after photothrombosis-induced permanent cerebral ischemia is not neuroprotective. Furthermore, sulforaphane did not influence on reactive gliosis nor did it limit the functional deficit when given as a single dose in the acute ischemic period or as repeated daily doses. Further studies in different models of cerebral ischemia, aimed at better understanding of the potential neuroprotective effects of sulforaphane and determining the optimal and clinically relevant time-point for administration, are warranted.
